# Accumulation of coumaric acid is a key factor in tobacco continuous cropping obstacles

**DOI:** 10.3389/fpls.2024.1477324

**Published:** 2024-10-28

**Authors:** Meng Jia, Xingsong Wang, Xuanquan Zhu, Yu Du, Peng Zhou, Ge Wang, Na Wang, Yuxiang Bai

**Affiliations:** College of Tobacco Science, Yunnan Agricultural University, Kunming, China

**Keywords:** tobacco, continuous cropping obstacles, phenolic acids, coumaric acid, allelopathic effects, soil microecology

## Abstract

**Introduction:**

Phenolic acids are believed to play a significant role in tobacco continuous cropping obstacles, but the strength and potential mechanisms of different phenolic acids remain unclear.

**Methods:**

This study evaluated the allelopathic effects of six phenolic acids that exhibited cumulative effects in our previous research. Different concentrations of phenolic acids with the strongest allelopathic effects were added to potting soil to explore their impacts on tobacco growth and physiological characteristics, as well as on soil chemical properties and microbial community structure.

**Results:**

The results showed that coumaric acid exhibited the strongest direct allelopathic effect. Exogenous coumaric acid significantly reduced soil pH and shifted the soil microbial community from bacteria-dominated to fungi-dominated. Simultaneously, the abundance of bacteria related to nutrient utilization (e.g., *Flavisolibacter*, *Methylobacterium*) and fungi related to disease resistance (e.g., *Fusicolla*, *Clonostachys*) gradually decreased, along with a reduction in soil catalase, urease, invertase, and acid phosphatase activities. Leaf MDA levels increased continuously with higher concentrations of coumaric acid, while the root resistance hormone (jasmonic acid and the jasmonate-isoleucine complex) levels show the opposite trend.

**Discussion:**

Coumaric acid may inhibit tobacco growth by influencing the physiological processes in tobacco plants directly and the broader soil microecological balance indirectly. This study provides theoretical guidance for precise mitigation of continuous cropping obstacles in future tobacco cultivation.

## 1 Introduction

The practice of continuous tobacco cropping has become increasingly prevalent in China (Su et al., 2022), with at least 30% of the annual planting area dedicated to this practice. This condition frequently leads to a series of monoculture-related challenges, including diminished physiological metabolic activity in plants, decreased yield and quality, and a higher incidence of pests and diseases (Tan et al., 2021). These issues pose severe limitations to the sustainable development of the tobacco industry. For a long time, numerous researchers have devoted their efforts to mitigating continuous cropping obstacles through various approaches, such as optimizing cropping systems (Liu et al., 2020a), applying soil amendments (Zhao et al., 2022), and employing soil fumigation (Xiong et al., 2023). Despite notable achievements, the widespread implementation of these strategies remains limited due to the prolonged duration required for observable benefits, soil heterogeneity, and significant environmental stressors.

Understanding the mechanisms underlying continuous cropping obstacles is crucial for the targeted mitigation of this issue. In recent years, research into soil autotoxic substances, especially phenolic acids, concerning continuous cropping obstacles has garnered considerable attention (Lin et al., 2023; Qiao et al., 2023). Phenolic acids are recognized as one of the most typical allelopathic secondary metabolites (Bao et al., 2022). They are released into the soil through various pathways, including leaching from plant leaves, decomposition of stems and other plant residues, and secretion from plant roots (Xie et al., 2017). These compounds are directly or indirectly implicated in the interaction between soil and plants, ultimately influencing plant growth and development (Hierro and Callaway, 2021). Continuous cropping of the same species, such as strawberries (Wang et al., 2019), poplars (Li et al., 2018), tobacco (Bai et al., 2019), and *Panax notoginseng* (Wang et al., 2022c), leads to the accumulation of phenolic acids in the soil (Shen et al., 2020), with their concentration tending to increase with the increasing duration of continuous cropping. Thus, the accumulation of phenolic acids is deemed a primary cause of continuous cropping obstacles (Chen et al., 2020). Previous studies have assessed the composition and allelopathic effects of phenolic acids in various crops, such as barnyard grass (Wang et al., 2022b), mango (Kato-Noguchi and Kurniadie, 2020), rice (Chi et al., 2013), and *Pinellia Rhizome* (Meng et al., 2022), highlighting the diversity of phenolic acids that cause continuous cropping obstacles across different crops and soils. However, our understanding of the allelopathic potency of different phenolic acids in tobacco-cultivated soil remains limited.

Research has established that the accumulation of phenolic acids in continuous cropping systems substantially influences the soil microecosystem, such as soil microbial communities, enzyme activities, and nutrient cycling (Guo et al., 2020; Lv et al., 2020). This accumulation deteriorates the soil microecological environment, consequently triggering continuous cropping obstacles (Yan et al., 2021). Soil microbes, highly sensitive to environmental changes, often serve as critical biological indicators of soil quality (Wang et al., 2022d). Additionally, interactions between rhizosphere microbes and root exudates are notably complex (Hussain et al., 2018). Previous research suggests that phenolic acids serve as an additional carbon (C) source and stimulate the growth of indigenous soil microbes, thereby altering the microbial community composition. Furthermore, compounds, phenolic acids, disrupt the balance between bacteria and fungi through selective inhibitory or stimulatory effects, leading to the accumulation of pathogenic microbes and a reduction in beneficial microbes (Ren et al., 2016). Although existing research has validated the pivotal role of soil microbes under phenolic acid stress, the complexity and diversity of soil environments still leave a gap in our understanding of how phenolic acids, particularly those with potent allelopathic effects, impact the soil microecological environment and contribute to the onset of continuous cropping obstacles.

Based on field-measured values of phenolic acids and curve estimation models from previous research, this study evaluated the allelopathic effects of various types and concentrations of phenolic acids *via* exogenous addition, identifying coumaric acid as the most potent. We further investigated the impact of varying concentrations of exogenously added coumaric acid on the physiological characteristics of tobacco and the microecological environment of tobacco-cultivated soil. These analyses sought to elucidate potential relationships among the soil environment, rhizosphere microbes, and tobacco plants under coumaric acid stress. Furthermore, this study aimed to clarify the mechanistic role of coumaric acid in tobacco continuous cropping obstacles and provide a theoretical foundation for targeted mitigation of continuous cropping obstacles in future agricultural practices.

## 2 Materials and methods

### 2.1 Soil sample collection

Soil samples were collected from a plot situated in Yaoguan Town, Shidian County, Baoshan City, Yunnan Province, China, which had never been subjected to tobacco cultivation (24°36′N, 99°14′E, elevation: 1820 m). The region exhibits an average annual temperature of 17° and an annual rainfall of 1120 mm. The soil is characterized by sandy loam. For the sampling methodology, we selected 60 sampling points, each spaced 2 m apart. At each designated point, one soil core (with a radius of 10 cm and a depth of 20 cm) was collected and subsequently sieved through a 2 mm mesh. The collected soil was thoroughly mixed; a portion thereof was analyzed to determine soil chemical properties and phenolic acid content (**Supplementary Table S1**), while the remainder was allocated for utilization in pot experiments.

### 2.2 Phenolic acid treatment

Our previous research identified eight phenolic acids in the soils of continuously cropped tobacco fields, six of which (phloroglucinol, coumaric acid, *p*-hydroxybenzoic acid, vanillic acid, vanillin, and ferulic acid) showed an accumulation trend with prolonged years of continuous cropping (Bai et al., 2019). Based on the curve estimation models fitted with environmental concentrations (**Table 1**), these six phenolic acids were subjected to three different stress concentration treatments corresponding to 8, 16, and 32 years of continuous cropping, along with a blank control (**Table 2**), amounting to 19 treatments in total. Each treatment was replicated in six pots, resulting in a total of 114 pots. The soil for the pot experiments was sterilized three times (each spaced 24 h apart) using a vertical pressure steam sterilizer (YXQ-LS-100G, Shanghai Boxun Industry & Commerce Co., Ltd., Medical Equipment Factory, Shanghai, China) at 121° (0.105 MPa) for 0.5 h. Following the final sterilization cycle, the soil was aseptically dispensed into 114 sterile plastic pots (top diameter of 12 cm, bottom diameter of 8.5 cm, and height of 10 cm) in a sterile environment, with each pot containing 500 g of soil, which was primed for phenolic acid treatment experiments.

**Table 1:** Environmental concentrations and optimal curve estimation models for phenolic acids with accumulation trends.

Phenolic Acids	Environmental concentrations across different years of continuous cropping (mg/kg)					Optimal curve estimation models	R^2^	P	Regression equation
	4 years	6 years	8 years	14 years	16 years				
Phloroglucinol	1.213	1.320	1.447	1.583	1.987	Growth	0.886	<0.01	y=EXP(0.065+0.035t)
Coumaric acid	0.050	0.049	0.058	0.063	0.134	Growth	0.785	<0.01	y=EXP(−3.384+0.070t)
*p*-hydroxybenzoic acid	0.127	0.187	0.122	0.279	0.467	Growth	0.844	<0.01	y=EXP(−2.589+0.106t)
Vanillic acid	0.073	0.123	0.089	0.145	0.278	Growth	0.706	<0.01	y=EXP(−2.863+0.087t)
Vanillin	0.090	0.148	0.128	0.166	0.291	Growth	0.792	<0.01	y=EXP(−2.529+0.071t)
Ferulic acid	0.032	0.044	0.122	0.132	0.318	Power	0.878	<0.01	$y=0.004t^{1.439}$

(y) represents the content of phenolic acids, and (t) represents the number of continuous cropping years of tobacco.

**Table 2:** Number and concentration of each phenolic acid treatment.

Phenolic Acids	Treatment ID	Added Concentration (mg/kg)
/	P0	0
Phloroglucinol	AM8	1.4120
	AM16	1.8682
	AM32	3.2707
Coumaric acid	BM8	0.0594
	BM16	0.1039
	BM32	0.3185
*p*-hydroxybenzoic acid	CM8	0.1753
	CM16	0.4094
	CM32	2.2322
Vanillic acid	DM8	0.1145
	DM16	0.2297
	DM32	0.9240
Vanillin	EM8	0.1407
	EM16	0.2483
	EM32	0.7734
Ferulic acid	FM8	0.0797
	FM16	0.2162
	FM32	0.5861

These specific phenolic acids, procured as standards from Sigma (St. Louis, MO, USA), were exogenously applied to the pots at predetermined concentrations. Simultaneously, 10 g of specialized tobacco fertilizer (N-P_2_O_5_-K_2_O=10-10-15) was uniformly introduced into each pot. Tobacco seedlings (variety: K326, aged 60 days) were subsequently transplanted, one per pot, and irrigated with 150 mL of sterile water. The pots were then placed in an artificially controlled climate chamber (LRH-8CO-GSJ; Shaoguan Taihong Medical Instrument Co., Ltd., Shaoguan, China), which had been sterilized with ultraviolet light and ozone for 6 h. The parameters for the chamber are detailed in **Supplementary Table S2**. The pots were randomly relocated once daily to avoid positional bias, and the seedlings were irrigated with 40 mL of sterile water every three days to maintain consistent moisture levels.

### 2.3 Coumaric acid application

Following the evaluation of allelopathic effects of phenolic acids, coumaric acid was chosen for subsequent exogenous addition experiments as it exerted the most pronounced allelopathic impact. Coumaric acid was administered at three stress concentrations corresponding to 8, 16, and 32 years of continuous cropping, along with one control (**Table 3**), resulting in four distinct treatments. Each treatment comprised 27 pots, amounting to 108 pots in total. Soil samples, not sterilized under high temperature and pressure, were transferred into 108 sterile plastic pots for experiments involving coumaric acid treatment. The other experimental treatments procedures were consistent with those used in the phenolic acid treatments.

**Table 3:** Number and concentration of coumaric acid treatments.

Phenolic Acids	Treatment ID	Added Concentration (mg/kg)
/	P0	0
Coumaric acid	BP8	0.0594
	BP16	0.1039
	BP32	0.3185

### 2.4 Assessment of tobacco growth and photosynthetic performance

Thirty days after phenolic acid treatment, tobacco plant heights were measured using the established agronomic trait survey method (YC/T 142-2010). The net photosynthesis rate (Pn) was quantified employing a LI-6400 Portable Photosynthesis System (Li-COR Inc., USA) between 09:00 am and 11:00 am, while soil and plant analyzer development (SPAD) indices were determined with a chlorophyll meter (SPAD-502 Plus, Japan) at the same time. Each treatment was replicated three times to ensure consistency.

Similarly, 30 days after coumaric acid treatment, plant height, stem girth, and leaf number were measured following the same procedures as delineated previously. Additionally, the roots were cleaned and weighed. Each of these measurements was also replicated three times to ascertain the reliability of the gathered data.

### 2.5 Evaluation of leaf enzyme activities and malondialdehyde levels

Thirty days after the application of phenolic acids, the activities of several key enzymes were quantified. Catalase (CAT) activity was determined using the ultraviolet absorption technique, polyphenol oxidase (PPO) was assessed through a colorimetric method, peroxidase (POD) activity was evaluated with the guaiacol method, and superoxide dismutase (SOD) was measured utilizing the nitro blue tetrazolium photoreduction method (Zhang et al., 2016). Each of these measurements was conducted in triplicate.

Subsequent to the application of coumaric acid, leaf MDA levels were quantified using the thiobarbituric acid reactive substances method (Li et al., 2019) at predetermined intervals: 10, 13, 16, 19, 21, 24, 27, and 30 days post-treatment. Each treatment was measured three times at each time point for replication.

### 2.6 Assessment of root and shoot weight, root vitality, and hormonal levels

Thirty days after the treatment with phenolic acids, tobacco seedlings were separated from their roots, which were then washed and weighed to determine root and shoot weight. Concurrently, the root-shoot ratio was calculated. The vitality of the roots was then evaluated through the triphenyltetrazolium chloride (TTC) assay, with each treatment subjected to triplicate testing.

Thirty days after the coumaric acid treatment, the concentrations of jasmonic acid (JA), abscisic acid (ABA) (Cheng et al., 2021), and the jasmonate-isoleucine complex (JA-Ile) (Liu et al., 2022) in the roots were determined. Consistency was maintained, with each parameter being measured in triplicate for every treatment.

### 2.7 Soil chemical properties, phenolic acids, and enzyme activity analysis

The collected baseline soil samples were analyzed to determine pH using the potentiometric method with a soil-to-water ratio of 2.5:1 (NY/T 1377-2007). Organic matter content was quantified using the potassium dichromate-sulfuric acid external heating method (NY/T 1121.6-2006). Total nitrogen, phosphorus, and potassium were measured using the semi-micro Kjeldahl, spectrophotometric, and flame photometric methods (NY/T 53-1987, NY/T 88-1988, NY/T 87-1988), respectively. Hydrolyzable nitrogen and available phosphorus were determined through the alkaline diffusion and ammonium acetate (CH_3_COONH_4_) extraction methods (LY/T 1229-1999, NY/T 1121.7-2014), and exchangeable potassium was assessed *via* ammonium acetate (NH_4_OAc) extraction flame photometry (NY/T 889-2004). The concentration of phenolic acids was determined through high-performance liquid chromatography (HPLC) (Bai et al., 2019). Each group of 20 soil cores was combined as a single replicate, with three replicates in total.

At 30 days after coumaric acid treatment, rhizosphere soil was collected using the root shaking method and analyzed for pH, organic matter content, and coumaric acid concentration using the same methods detailed previously. Enzymatic activities in the soil, such as CAT, urease (URE), invertase (INV), and acid phosphatase (ACP), were quantified using the potassium permanganate (KMnO_4_) titration, indophenol colorimetric assay, 3,5-dinitrosalicylic acid (DNS) colorimetric assay, and disodium phenyl phosphate colorimetry, respectively (Lu et al., 2023). Each set of measurements was obtained from paired plants and replicated three times to ensure the robustness of the data collected.

### 2.8 Determination of soil microbial communities

Thirty days after coumaric acid treatment, rhizosphere soil was collected using the root shaking method. Soil from every two plants was aggregated to compose one replicate, with each treatment replicated three times.

The total DNA from the samples was extracted using the HiPure Soil DNA kit (Magen, Guangzhou, China). Subsequently, the quality of the genomic DNA was assessed *via* 1% agarose gel electrophoresis. Following centrifugation, the samples were diluted with sterile water to achieve a concentration of 1 ng/µL.

The PCR amplification conditions were as follows: an initial denaturation at 95°C for 5 minutes, followed by 30 cycles of 95°C for 1 minute, 60°C for 1 minute, and 72°C for 1 minute, with a final extension at 72°C for 7 minutes using specific primers. The 16S V4 region amplified using primers 515F-806R (515F:GTGYCAGCMGCCGCGGTAA, 806R:GGACTACHVGGGTATCTAAT), and the ITS1 region using primers 1737F-2043R (1737F:GGAAGTAAAAGTCGTAACAAGG, 2043R:GCTGCGTTCTTCATCGATGC). The amplification system consisted of a 50 μL reaction mixture containing: 10 μL of 5×Q5^®^ Reaction Buffer, 10 μL of 5×Q5^®^ High GC Enhancer, 1.5 μL of 2.5 mM dNTPs, 1.5 μL of each primer (10 μM), 0.2 μL of Q5^®^ High-Fidelity DNA Polymerase, and 50 ng of template DNA. All PCR-related reagents were sourced from New England Biolabs (USA).

Amplicons were collected from a 2% agarose gel and purified using the AxyPrep DNA Gel Extraction Kit (Axygen Biosciences, Union City, CA, USA) according to the manufacturer’s instructions. The purified amplicons were then quantified using the ABI StepOnePlus real-time PCR system (Life Technologies, Foster City, USA). Subsequently, the purified amplicons were prepared for Illumina paired-end sequencing.

The raw data were assembled and subsequently filtered to eliminate any inconsistencies. These data underwent clustering and were taxonomically analyzed based on operational taxonomic units (OTUs). Initial analysis results were obtained by integrating the raw data.

### 2.9 Statistical analysis

The response index (RI) and physiological parameters for each treatment’s effect on tobacco growth were calculated using either Equation 1 or 2 to quantify the allelopathic effect.


(1) RI=1−C/T   (T≥C)

(2) RI=T/C−1   (T<C)

Here, *C* represents the control value, and *T* represents the treatment value. An RI > 0 denotes a stimulatory effect, while an RI< 0 indicates an inhibitory effect. The absolute value of RI reflects the intensity of the effect, whether stimulatory or inhibitory. The synthetic allelopathic effect index (SE) was evaluated using the mean of the RI values (Wang et al., 2022a).

The experimental data were initially processed using Microsoft Excel 2023 and GraphPad Prism 8.0.2. Subsequently, R (version 3.4.3) was used to perform significance analysis on plant physiological characteristics, soil chemical properties, soil enzyme activity, and plant growth. Prior to significance testing, the Shapiro-Wilk test was used to assess normality, and the F-test was applied to analyze the homogeneity of variances. Microbial data were analyzed using the Omicshare platform (https://www.omicshare.com/). Principal coordinate analysis (PCoA) was conducted by calculating Bray–Curtis distances among the samples, and visualization was also performed using the same platform. Additionally, based on the PCoA results, bacterial and fungal distance matrices were exported, and the Mantel function from the “vegan” package in R was used to calculate the correlation between the two matrices, which characterizes the interaction between bacterial and fungal community structures. Lefse differential species analysis was performed using the “microeco” package in R, with the LDA score set to 4.0 for bacteria and 3.0 for fungi. Microbial network analysis was conducted using OTUs with a relative abundance greater than 1% across different treatments. Spearman correlation coefficients between OTUs were calculated using R, and effective links were considered to exist between nodes when p<0.01 and |r|≥0.9. The resulting networks were visualized and analyzed in Gephi (version 0.9) to compare topological features between treatments. Bacterial function prediction was annotated using the KEGG Pathway in PICRUSt2, while fungal function prediction was annotated using FUNGuild. Structural Equation Modeling (SEM) was performed using Partial Least Squares Path Modeling (PLS-PM) with the “plspm” package in R, evaluating the relationships among coumaric acid, soil chemical properties, plant stress indicators, root hormones, the microbiome, and plant growth indicators. Path coefficients and R² values were estimated and validated using the bootstrap method (1,000 iterations). To ensure the model’s reliability, latent variables with a loading value<0.8 were excluded. Finally, the model’s overall predictive performance was evaluated using the Goodness of Fit (GOF) index.

## 3 Results

### 3.1 Evaluation of the allelopathic effects of phenolic acids on tobacco growth

**Figure 1:**
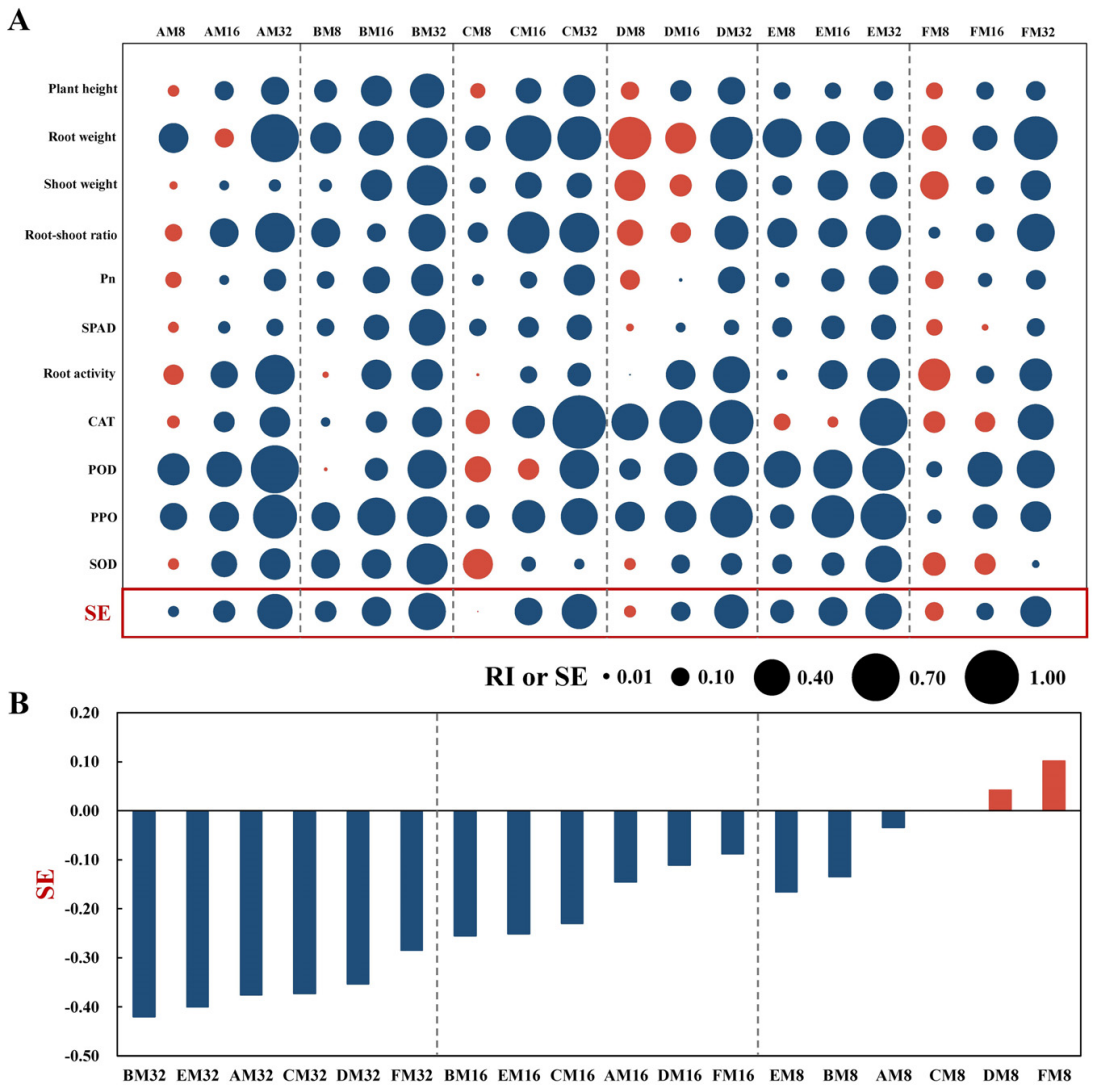
Allelopathic effects of phenolic acids on tobacco growth and their synthetic allelopathic effects. **(A)** Bubble chart illustrating the response index (RI) and synthetic allelopathic effect index (SE) for growth and physiological parameters across various treatments. Blue bubbles signify inhibitory effects (RI or SE< 0), while red bubbles indicate stimulatory effects (RI or SE > 0), with the bubble size corresponding to the magnitude of the absolute value. **(B)** SE of different phenolic acids on tobacco growth under identical concentrations.

Relative to the control (**Figure 1A**), while certain growth and physiological indices, such as root weight (RI_max_=0.548), root activity (RI_max_=0.310) and shoot weight (RI_max_=0.287), were enhanced under low to moderate concentrations of phenolic acid treatments, an overarching trend of inhibition (RI< 0) was observed with gradually increasing phenolic acid concentrations, and the RI for each index under high- concentration treatments ranged from -0.892 to -0.016. This inhibitory trend was also noted in the overall SE, where the absolute value of SE increased by over 140% in the high-concentration treatments compared to the low-concentration treatments. Further analysis (**Figure 1B**) revealed that at low concentrations, vanillic acid and ferulic acid promoted tobacco growth, whereas other phenolic acids conferred inhibitory effects, with vanillin exhibiting the strongest inhibition, and the absolute value of SE was 0.031 higher than that of coumaric acid. At medium and high concentrations, all six phenolic acids exhibited negative synthetic allelopathic indices, with coumaric acid showing the most prominent suppressive effect. The absolute values of SE for coumaric acid were 0.004 and 0.020 higher than those of vanillin, respectively. Consequently, coumaric acid was selected for further comprehensive studies due to its strongest direct allelopathic effects, to investigate its mechanisms of action in the soil environment of tobacco cultivation.

### 3.2 Impact of coumaric acid on tobacco growth and physiological responses

As the concentration of coumaric acid increased, the inhibitory effect on tobacco growth progressively intensified (**Supplementary Table S3**). Correspondingly, the MDA content in tobacco leaves exhibited a consistent upward trend across different time intervals (**Supplementary Figure S1A**). The rate of change in MDA content peaked between days 19 and 21 before subsequently declining (**Supplementary Figure S1B**). Moreover, coumaric acid treatment notably reduced the levels of JA and JA-Ile in the roots, with high-concentration treatments resulting in reductions exceeding 800% compared to the control. Conversely, ABA levels in the treatment groups are higher than those in the control group, but there are no significant differences among the treatments. (**Supplementary Figures S1C–E**).

### 3.3 Observations of the soil microecological environment and residual coumaric acid

With increasing concentrations of coumaric acid, reductions were observed in soil pH, organic matter content, and the activities of CAT, ACP, URE, and INV. Among these, CAT showed the greatest decrease, with reductions of 40.91%, 45.48% and 52.69% at the three concentrations, respectively, compared to the control. Concurrently, the residual concentration of coumaric acid in the soil progressively increased. Notably, even the control soil samples contained detectable levels of coumaric acid, with a concentration of 0.008 mg/kg. (**Supplementary Figure S2**).

### 3.4 Analysis of soil microbial diversity changes under coumaric acid stress

**Figure 2:**
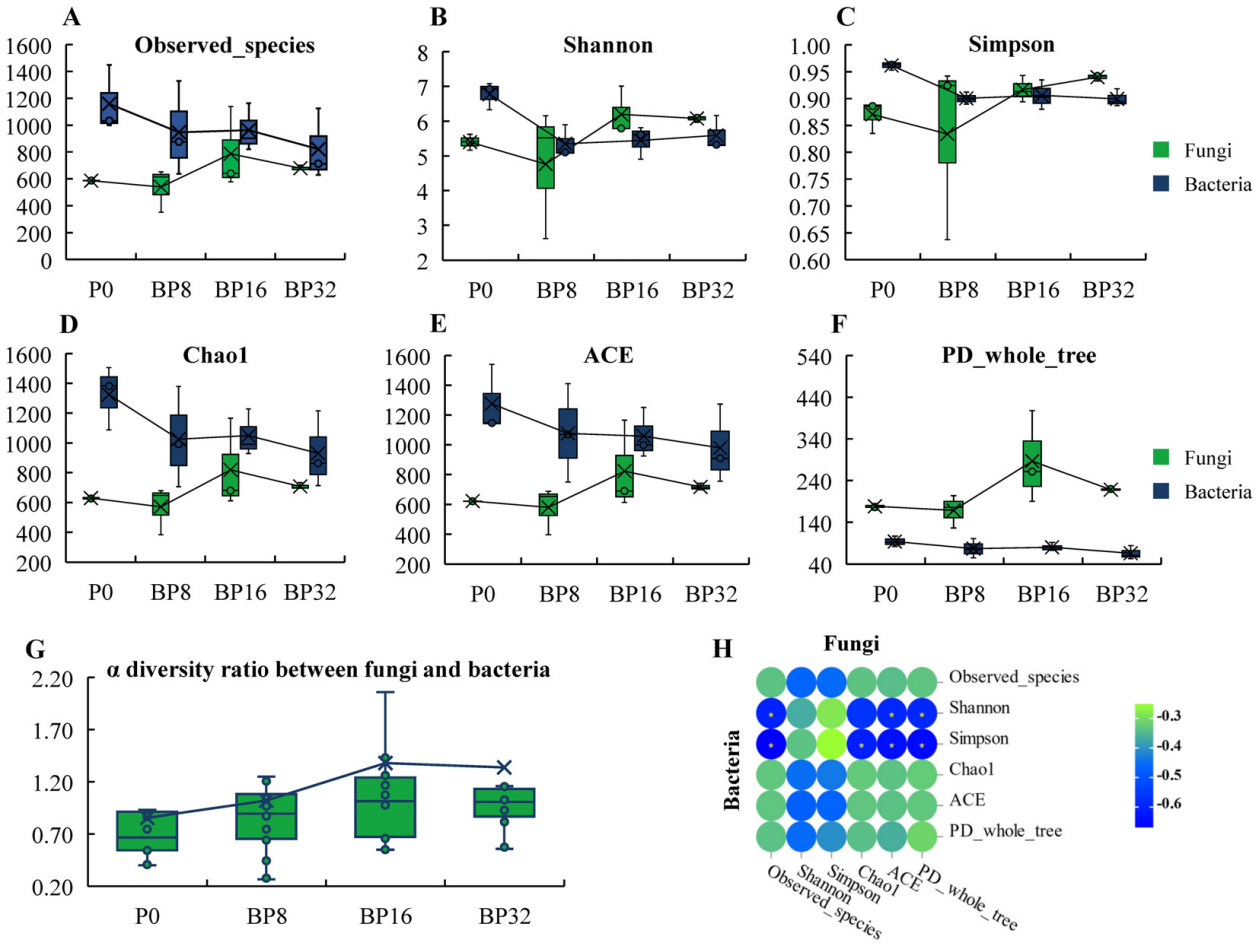
Analysis of soil microbial diversity under coumaric acid stress. **(A–F)** Charts illustrating the α diversity of bacterial and fungal communities. **(G)** Graph depicting the α diversity ratio between fungi and bacteria. **(H)** Heatmap of correlations between the α diversities of bacteria and fungi.

With rising levels of coumaric acid, a discernible decline was observed in the α diversity of soil bacteria, while fungal diversity exhibited an increasing pattern (**Figures 2A–F**). After coumaric acid treatment, the bacterial α diversity index decreased by 5.63% to 29.78%, with the Chao1 index showing the greatest reduction. Conversely, after treatment with medium and high concentrations of coumaric acid, the fungal α-diversity index increased by 5.36% to 60.47%, with the highest increase observed in the PD_whole_tree index. Subsequent analysis revealed a progressive increase in the ratio of fungal to bacterial α diversity (**Figure 2G**), reaching a peak after 16 years of continuous cropping treatment, with a 62.35% increase compared to the control. Correlations among bacterial and fungal α diversity indices were predominantly negative (**Figure 2H**), affirming that coumaric acid stress induced a transition from bacterial to fungal dominance within the soil microbial community utilized for tobacco cultivation.

**Figure 3:**
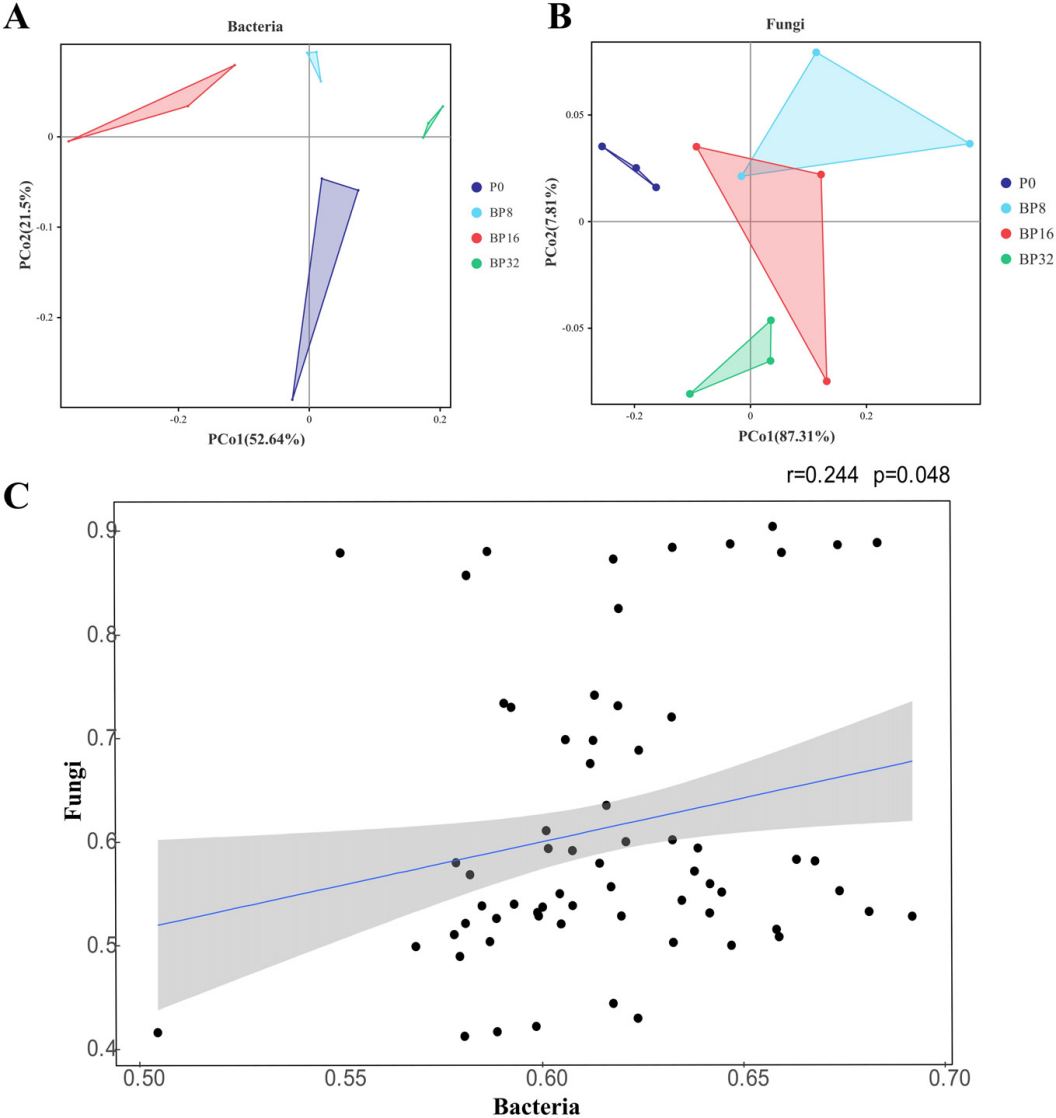
Effects of coumaric acid on the structure of soil bacterial and fungal communities. **(A)** and **(B)** represent the differences in bacterial and fungal community structures between treatments, respectively. **(C)** shows the correlation between bacterial and fungal community structures, where the blue line indicates the linear fit, and the gray area represents the 95% confidence interval.

PCoA revealed progressive alterations in the community structures of soil bacteria and fungi with increasing coumaric acid concentrations. For bacteria, the community structure of the control group was more similar to that of the low-concentration treatments, and markedly different from the medium- and high-concentration treatments (**Figure 3A**). Conversely, fungal communities in the control were more akin to those in the medium concentration treatments, but differed significantly from the low- and high-concentration treatments (**Figure 3B**). Mantel test results based on the microbial community structure highlighted inconsistent differences between bacterial and fungal structures at the OTU level (*r* = 0.244, *p* = 0.048), suggesting different response mechanisms to coumaric acid stress between soil bacteria and fungi (**Figure 3C**).

### 3.5 Analysis of soil microbial community structure

**Figure 4:**
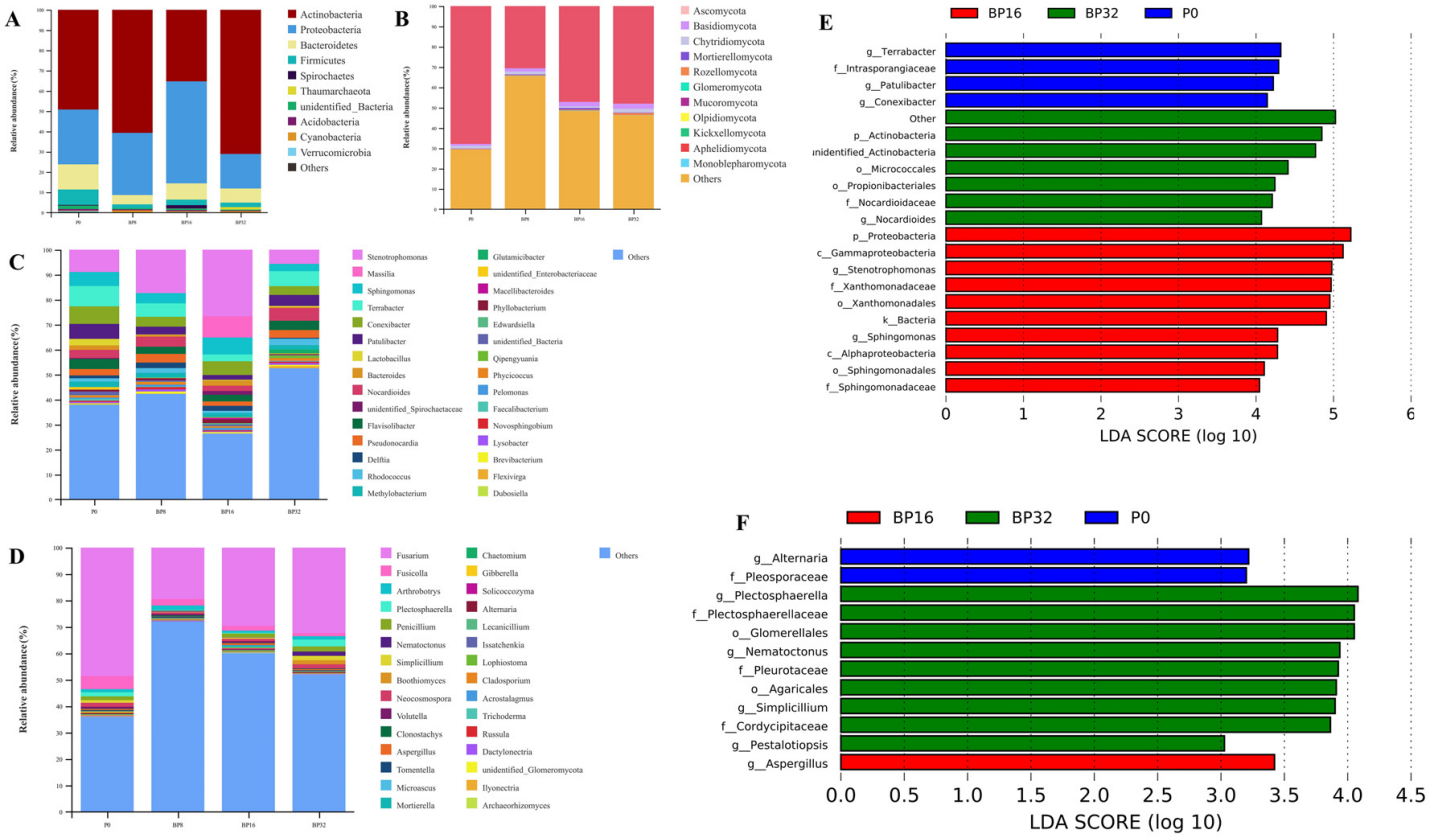
Comparative analysis of bacterial and fungal communities in samples P0, BP8, BP16, and BP32. **(A)** Relative abundance of bacterial phyla. **(B)** Relative abundance of fungal phyla. **(C)** Relative abundance of bacterial genera. **(D)** Relative abundance of fungal genera. **(E)** Latent Dirichlet allocation (LDA) bar graph based on LEfSe analysis representing the bacterial microbiome with LDA scores above 4.0. **(F)** LDA histogram based on LEfSe analysis, representing the fungal microbiome with LDA scores above 3.0. The LDA scores highlight the statistical and biological differences among the clades.

With increasing concentrations of coumaric acid, notable shifts were observed in the soil microbial community. Regarding soil bacteria, the primary phyla exhibiting an average relative abundance >0.5% were *Actinobacteria*, *Proteobacteria*, *Bacteroidetes*, and *Firmicutes*, which collectively accounted for 95.68% of the total sequencing data (**Figure 4A**). The relative abundance of *Actinobacteria* demonstrated an escalating trend, while *Proteobacteria* initially exhibited an increase followed by a subsequent decline. Conversely, *Bacteroidetes* and *Firmicutes* showed a decline in relative abundance. In soil fungi, phyla with an average relative abundance >0.1% included *Ascomycota*, *Basidiomycota*, and *Chytridiomycota*, accounting for 51.60% of the total sequencing data (**Figure 4B**). The relative abundance of *Ascomycota* decreased, whereas that of *Basidiomycota* and *Chytridiomycota* increased with rising levels of coumaric acid.

Among bacterial genera with a relative abundance over 0.1%, *Massilia* and *Glutamicibacter* demonstrated increased relative abundance, while *Terrabacter*, *Conexibacter*, *Flavisolibacter*, *Methylobacterium*, and *Faecalibacterium* experienced decreases (**Figure 4C**). In soil fungal genera with a relative abundance over 0.1%, the genus *Boothiomyces* displayed an increasing trend in relative abundance, whereas *Fusarium*, *Fusicolla*, *Clonostachys*, *Gibberella*, *Lecanicillium*, *Cladosporium*, and *Ilyonectria* exhibited declining trends (**Figure 4D**).

The LEfSe analysis was utilized to characterize key microbial differences between treatments. The results suggested that, at the genus level in soil bacteria, *Stenotrophomonas* and *Sphingomonas* were enriched in medium-concentration treatments, whereas *Nocardiodes* was enriched in high-concentration treatments (**Figure 4E**). In the fungal community, *Aspergillus* was enriched in medium-concentration treatments, while *Plectosphaerella*, *Nematoctonus*, *Simplicillium*, and *Pestalotiopsis* exhibited enrichment in high-concentration treatments (**Figure 4F**). These observations highlight that coumaric acid influences key microbial groups, leading to alterations in the structure of soil bacterial and fungal communities.

### 3.6 Topological analysis of soil microbial co-occurrence networks

**Figure 5:**
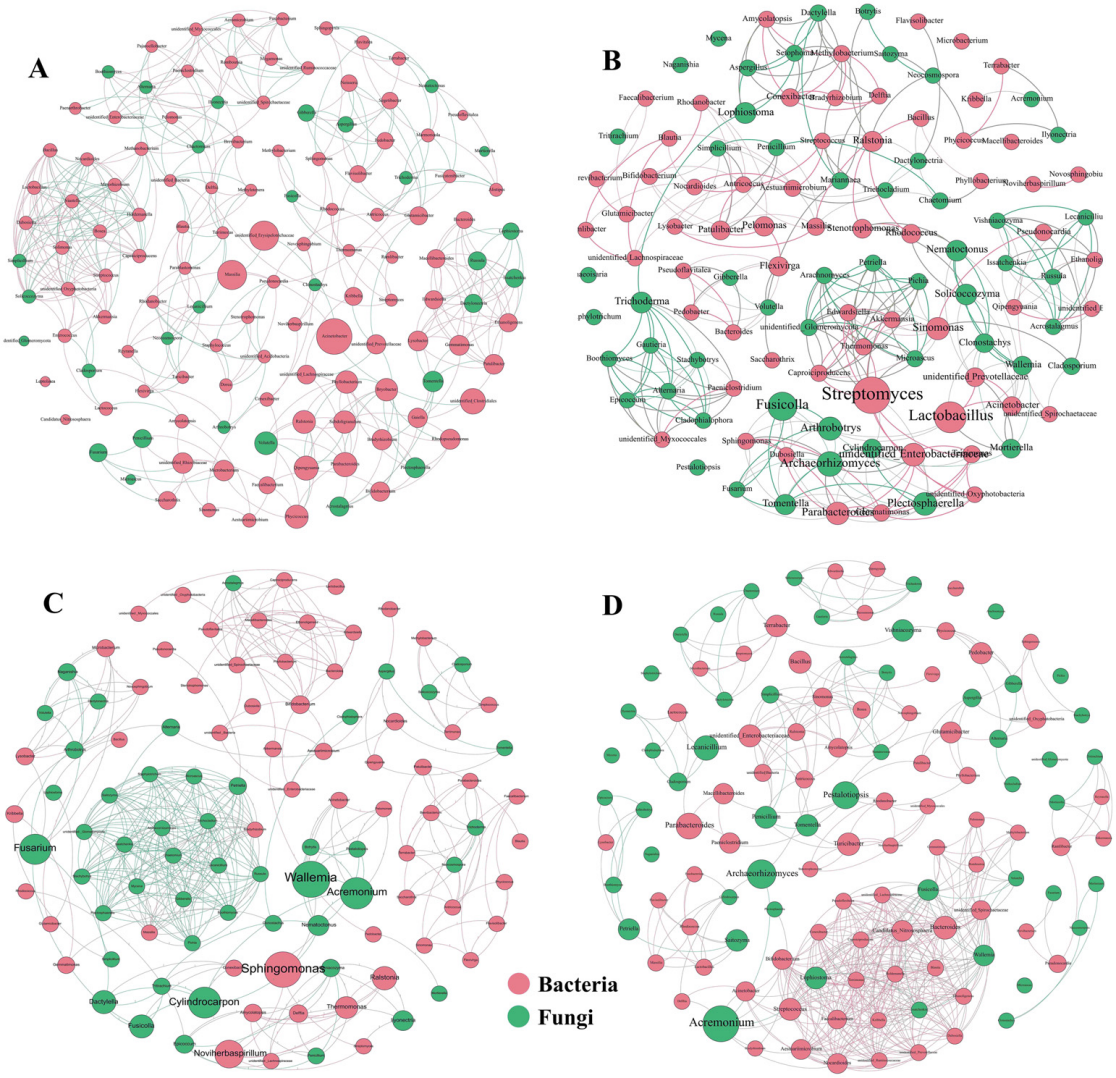
Co-occurrence network topology of soil bacteria and fungi across varying concentrations of exogenous coumaric acid: P0 **(A)**, BP8 **(B)**, BP16 **(C)**, and BP32 **(D)**. Bacteria are depicted as red circles, while fungi are depicted as green circles. Red and green lines denote positive and negative inter-genus correlations, respectively.

Coumaric acid stress led to significant changes in the complexity of the soil microbial network compared to the control (**Figures 5A–D**). With increasing coumaric acid concentration, the network diameter and average path length tended to decrease, while the number of connected components and the average clustering coefficient increased. In addition, coumaric acid treatment resulted in a decrease in the proportion of bacteria and an increase in the proportion of fungi in the soil (**Table 4**).

**Table 4:** Effects of exogenous coumaric acid on the topological characteristics of the co-occurrence network of soil bacteria and fungi.

Co-occurrence network topological properties	P0	BP8	BP16	BP32
Average Degree	6.48	5.60	7.14	7.87
Diameter	27	13	12	8
Density	0.051	0.049	0.067	0.065
Weakly Connected Components	10	17	11	18
Average Clustering Coefficient	0.748	0.817	0.82	0.794
Average Path length	8.597	3.505	3.223	2.178
Modularity	0.819	0.844	0.740	0.794
Bacterial proportion (%)	77.52	53.91	55.14	58.20
Fungal proportion (%)	22.48	46.09	44.86	41.80

Importantly, key microbes identified through LEfSe analysis, such as *Stenotrophomonas*, *Sphingomonas*, *Aspergillus*, *Nocardiodes*, *Plectosphaerella*, *Nematoctonus*, *Simplicillium*, and *Pestalotiopsis*, were also central nodes in the microbial co-occurrence network topology.

### 3.7 Predictive analysis of soil microbial functions

**Figure 6:**
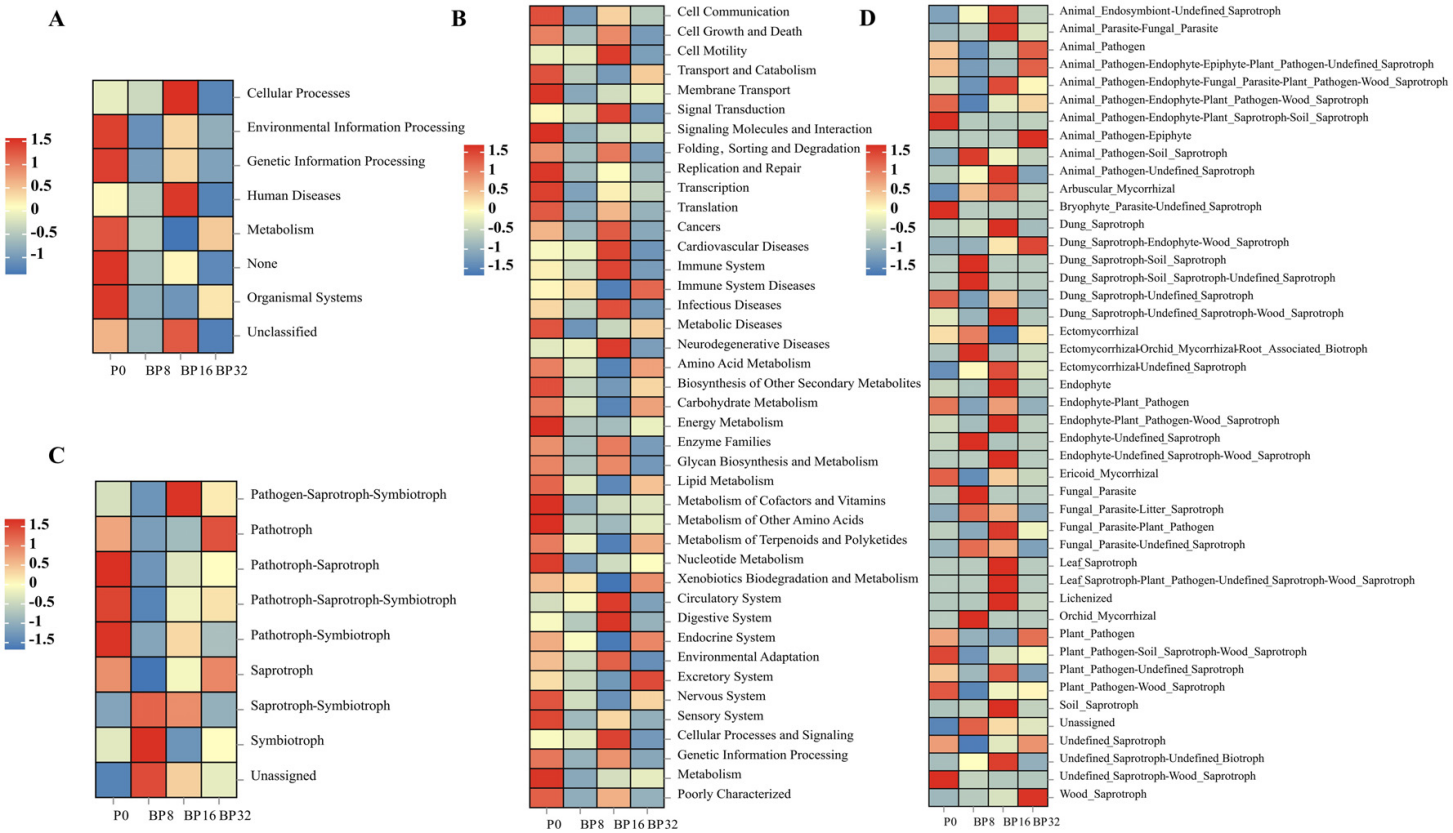
Heatmap illustrating the KEGG functional diversity of soil bacteria at the first **(A)** and second **(B)** hierarchical levels across different treatments. Color intensity corresponds to differences from the mean expression of metabolic functions under different treatments. **(C, D)** Predicted functions of fungal communities assessed using FUNGuild.

Based on the PICRUSt algorithm, we analyzed the functional profiles of soil bacterial communities exposed to varying coumaric acid treatments. At the first hierarchical level, significant alterations were noted in areas such as environmental information processing, genetic information processing, metabolism, and organismal systems (**Figure 6A**). At the second hierarchical level, functions such as membrane transport, amino acid metabolism, carbohydrate metabolism, replication and repair, energy metabolism, lipid metabolism, metabolism of cofactors and vitamins, and nucleotide metabolism were diminished with increasing concentrations of coumaric acid (**Figure 6B**).

Functional predictions of soil fungal communities using FUNGuild revealed three trophic modes (pathotrophs, saprotrophs, and symbiotrophs) and five mixed trophic modes (pathogen-saprotroph-symbiotroph, pathogen-saprotroph-symbiotroph, pathotroph-saprotroph-symbiotroph, pathotroph-symbiotroph, and saprotroph-symbiotroph) in the fungal community. The dominant functional group in soils treated with exogenous coumaric acid was saprotroph-symbiotroph (**Figure 6C**). Furthermore, with rising concentrations of coumaric acid, functions such as “Dung_Saprotroph-Endophyte-Wood_Saprotroph” and “Wood_Saprotroph” were progressively enhanced (**Figure 6D**).

### 3.8 Structural equation model of interactions between tobacco growth and the soil microenvironment

**Figure 7:**
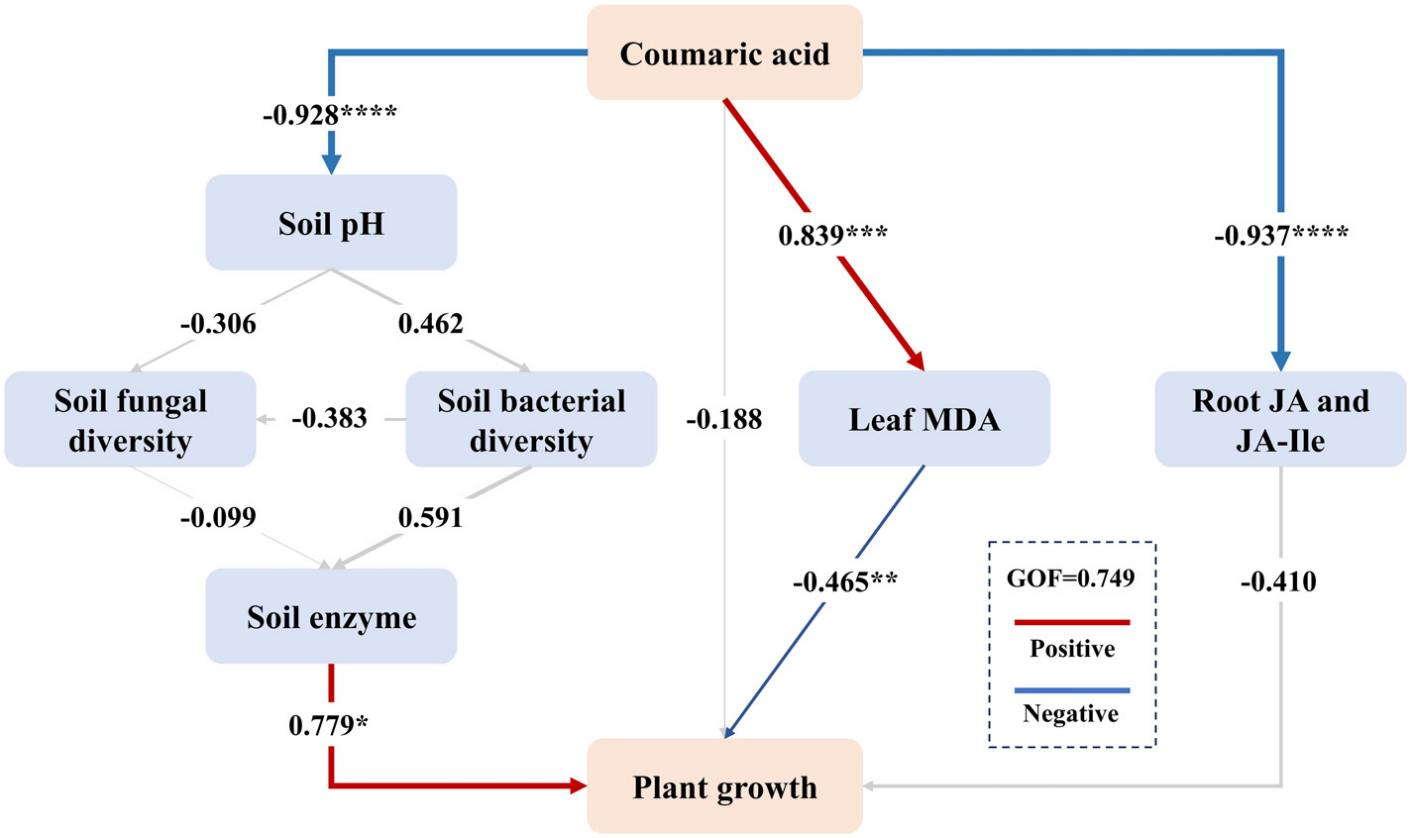
The Partial Least Squares Path Modeling (PLS-PM) was used to analyze the relationships between coumaric acid, soil pH, soil microorganisms, soil enzyme activity, leaf MDA, plant hormones, and tobacco growth. “Coumaric acid” represents the amount of coumaric acid added. “Soil fungal diversity” includes the Simpson, ACE, and PD_whole_tree indices, while “Soil bacterial diversity” includes the Shannon, Chao1, and PD_whole_tree indices. Soil enzymes include CAT, URE, ACP, and INV. “Plant growth” includes plant height, stem girth, leaf number, and root weight. Path coefficients were estimated using 1,000 bootstraps. Positive and negative effects are indicated by red and blue arrows, respectively, while non-significant path coefficients are shown with gray lines. * indicates p< 0.05, ** indicates p< 0.01, *** indicates p< 0.001, and **** indicates p< 0.0001. The model was evaluated using the Goodness of Fit (GOF) value.

Based on SEM, we fitted a model to explore the relationships among the addition of coumaric acid, soil pH, bacterial and fungal α diversity indices, soil enzyme activities, and the physiological and growth characteristics of tobacco plants (**Figure 7**). The model demonstrated a good fit (goodness-of-fit (GOF)= 0.749). The results indicated that the addition of coumaric acid significantly reduced soil pH (path coefficient = -0.928). Soil pH was positively correlated with bacterial diversity (path coefficient = 0.462) and negatively correlated with fungal diversity (path coefficient = -0.306). Bacterial diversity was positively associated with soil enzyme activity, while fungal diversity exhibited an opposite trend. Furthermore, soil enzyme activity was positively correlated with tobacco growth (path coefficient = 0.779). Conversely, the introduction of coumaric acid significantly stimulated stress indicators, ultimately suppressing tobacco growth. Overall, coumaric acid indirectly restricted tobacco growth by influencing soil pH, bacterial and fungal diversity, soil enzyme activity, and directly through its impact on MDA in leaves.

## 4 Discussion

### 4.1 Potent allelopathic effects of coumaric acid on tobacco plants

Phenolic acids exert allelopathic effects by influencing the growth of plant roots (Wang et al., 2022b), root vitality (Wang et al., 2019), photosynthesis in leaves (Lu et al., 2018), and antioxidant enzyme activities (Weremczuk-Jezyna et al., 2021), ultimately impeding overall plant growth and development (Jin et al., 2020). These studies, however, employed exogenous additions in natural soils and did not verify the direct allelopathic actions of phenolic acids. In our controlled environment with sterilized soil, we examined the direct impacts of six phenolic acids on the growth and development of tobacco plants and assessed the intensity of allelopathic effects of these acids at different concentrations using the RI and the SE. Our results showed variations in allelopathic effects across different types and concentrations of phenolic acids. Phloroglucinol, *p*-hydroxybenzoic acid, vanillic acid, and ferulic acid exhibited a “low stimulation-high inhibition” effect, whereas coumaric acid and vanillin displayed an enhanced inhibitory effect with rising concentrations. Moreover, in this study, as concentrations of coumaric acid and vanillin increased, significant reductions in SPAD values and Pn values were observed in tobacco leaves. This outcome is likely ascribed to phenolic acids affecting chlorophyll synthesis, thereby inhibiting photosynthesis (Ghimire et al., 2020; Fu et al., 2023). Given the central role of chlorophyll in photosynthesis and its susceptibility to reactive oxygen species (ROS) damage, antioxidant enzymes are crucial for sustaining normal plant growth under stress conditions (Sun et al., 2022). We found that increasing concentrations of coumaric acid and vanillic acid intensified the inhibitory effect on antioxidant enzyme activities in leaves, indicating that phenolic acids could cause oxidative damage to the antioxidant enzyme system of tobacco plants, thereby suppressing growth and development. Moreover, our evaluation of synthetic allelopathic effects revealed coumaric acid as exerting the strongest allelopathic action. This finding leads to the conclusion that phenolic acids can directly toxify and inhibit the growth of tobacco plants, with the magnitude of allelopathic effects contingent upon the specific type and concentration of phenolic acids.

### 4.2 Coumaric acid stress inhibits tobacco growth and affects its physiological characteristics

The physiological functions of plants are closely associated with the metabolism of ROS in plant cells (Tang et al., 2017). The MDA content is indicative of the extent of lipid peroxidation damage to plant cell membranes. Our investigations illuminated that as concentrations of coumaric acid increased, the MDA content in tobacco leaves also rose correspondingly at various intervals, with the most significant alteration observed on the 19th day of treatment. SEM further corroborated a significant positive correlation between coumaric acid levels and MDA content. This increase in MDA content might be attributed to intensified ROS production under coumaric acid stress, which disrupts C assimilation processes in leaves, thereby initiating lipid peroxidation. Consequently, this reaction increases cell membrane permeability, leading to disruptions in cellular physiological functions (Zhang et al., 2019), consequently suppressing overall plant growth.

Plants respond to stress by modulating endogenous hormone levels to adapt to challenging environmental conditions (Agarwal et al., 2021), such as a rapid increase in ABA levels (Li et al., 2022). Similarly, in this study, ABA levels in the roots of tobacco plants subjected to low, medium, and high concentrations of coumaric acid were higher than those in the control group. Moreover, we observed a notable decrease in the contents of JA and JA-lle with increasing coumaric acid concentrations. JA compounds, including JA and JA-Ile, serve as signaling molecules involved in plant responses to abiotic stress, with linolenic acid, a precursor of JA, regulating K^+^ channels in guard cell plasma membranes, thereby influencing stomatal activity. Hence, we speculate that under coumaric acid stress, JA compounds regulate K^+^ channels in the plasma membranes of tobacco cells and affect stomatal activity, leading to suppressed growth and diminished stress resilience in tobacco plants. This inhibitory effect might also be attributed to the accumulation of coumaric acid and resultant alterations in the soil microenvironment.

### 4.3 The accumulation of coumaric acid leads to soil acidification, alters microbial community structure, and reduces soil enzyme activity

The phenomenon of phenolic acid accumulation in soils subjected to continuous cropping has been extensively documented in various studies (Lin et al., 2023; Qiao et al., 2023). Our research also elucidated detectable levels of coumaric acid in soil not initially treated with it following tobacco cultivation, indicating that tobacco plants synthesize and secrete coumaric acid during their growth. Furthermore, in soils treated with supplementary coumaric acid, residual levels of this compound coumaricincreased proportionally to the concentration applied. Accordingly, it is speculated that although soil microbes degrade and utilize phenolic acids as a C source (Zwetsloot et al., 2020), persistent alterations in soil conditions, coupled with the biosynthesis and secretion by tobacco plants, contribute to the observed accumulation of phenolic acids in the soil.

The impact of phenolic acids accumulation on soil physicochemical properties should not be overlooked (Ni et al., 2021). In our study, the exogenous addition of coumaric acid led to a decrease in soil pH in the tobacco planting environment, with a significant drop observed starting at the medium concentration of coumaric acid treatment. This indicates that short-term accumulation of coumaric acid does not rapidly acidify the soil, but long-term accumulation greatly increases the risk of soil acidification, as confirmed in continuous cropping soils in the field (Xie et al., 2017). Several studies have demonstrated that soil pH is a key factor influencing the soil micro-ecological environment (Naz et al., 2022; Zhong et al., 2023), and fluctuations in its value may indicate directional changes in the structure of soil microbial communities.

Soil microorganisms play a vital role in soil material and energy metabolism. Our study found that soil bacterial α-diversity decreased with increasing concentrations of coumaric acid, while fungal α-diversity and the ratio of fungal to bacterial α-diversity exhibited the opposite trend. Additionally, bacterial and fungal α-diversities were generally negatively correlated. Previous research has shown that phenolic acids, as organic compounds, can alter microbial community structure by providing carbon sources and energy substrates to microorganisms (Chen et al., 2018). Furthermore, we noted that previous study on acidified soils demonstrated that bacterial α-diversity decreases continuously as soil pH declines (Wang et al., 2022e). On the other hand, acidification creates a more favorable environment for pathogens, which are predominantly fungal, to thrive (Tagawa et al., 2010). Therefore, we hypothesize that coumaric acid may directly, or indirectly through its effect on soil pH, alter soil microbial diversity, shifting the community structure from bacteria-dominated to fungi-dominated. This finding also partially explains the shift observed under continuous cropping conditions in the field (Liu et al., 2020b; Chen et al., 2022).

Coumaric acid stress altered the relative abundance of soil microbial communities. As the concentration of coumaric acid increased, the relative abundance of bacterial genera such as *Terrabacter*, *Conexibacter*, *Flavisolibacter*, *Methylobacterium*, and fungal genera such as *Fusarium*, *Fusicolla*, *Clonostachys*, and *Gibberella* gradually decreased. Previous studies have shown that *Conexibacter* contributes to promoting the soil carbon cycle (Deng et al., 2015), *Flavisolibacter* aids plant growth under abiotic stress by regulating rhizosphere soil nutrient structure (Lin et al., 2021), and *Methylobacterium* is a nitrogen-fixing microorganism (Zhu et al., 2023). *Fusicolla* can kill pathogenic fungi by disrupting cell membrane integrity and altering hyphal morphology (Li et al., 2021), while *Clonostachys* resists pathogenic fungi by secreting cell wall-degrading enzymes and producing antifungal secondary metabolites (Sun et al., 2020). Based on this analysis, we infer that coumaric acid may affect tobacco growth by reducing the abundance of bacteria related to nutrient utilization and fungi involved in disease resistance. LEfSe analysis revealed that under medium-concentration coumaric acid treatment, the bacterial genera *Stenotrophomonas* and *Sphingomonas*, and the fungal genus *Aspergillus*, were enriched. *Sphingomonas* can regulate plant growth under abiotic stress by producing plant growth hormones (Asaf et al., 2020), while *Aspergillus* is involved in the remediation of both biotic and abiotic soil contamination (Kumar et al., 2021). In contrast, fungal genera *Plectosphaerella*, *Nematoctonus*, *Simplicillium*, and *Pestalotiopsis* were enriched under high-concentration treatment, with *Plectosphaerella* (Gao et al., 2023) and *Pestalotiopsis* (Zhang et al., 2024) commonly being pathogenic fungi. Interestingly, these functionally distinct genera also served as central nodes in the co-occurrence networks. Based on this, we speculate that soil under medium-concentration treatment develops some resistance, while high-concentration treatment is dominated by pathogenic fungi.

Soil enzyme activity is closely related to soil health and nutrient availability. For example, CAT catalyzes the decomposition of hydrogen peroxide (Wojewódzki et al., 2022), protecting organisms from its toxic effects. URE specifically catalyzes the hydrolysis of urea and is directly related to soil nitrogen content (Coelho et al., 2022). In our study, we found that the activities of enzymes such as URE, ACP, CAT, and INV significantly decreased with increasing concentrations of coumaric acid. We observed that in continuous cropping soils, the available nitrogen, phosphorus, and potassium contents, along with urease and sucrase activities, also decreased. This may be due to the accumulation of phenolic compounds during continuous cropping, which could form hydrogen bonds with soil enzymes, reducing their activity (Ni et al., 2021). Additionally, studies have shown that the accumulation of pathogens and toxic substances in the soil can directly reduce soil enzyme activity (Bai et al., 2019). On the other hand, through bacterial function prediction, we found that several metabolic functions, such as amino acid metabolism, carbohydrate metabolism, and energy metabolism, weakened as the concentration of coumaric acid increased. This suggests that the lack of sufficient substrates for enzymatic reactions may contribute to the decrease in soil enzyme activity. Based on these findings, we can infer that coumaric acid stress may reduce soil enzyme activity by directly affecting the enzymes, reacting with them, or altering the structure and function of bacterial communities.

### 4.4 Identified key factors for determining tobacco growth affected by coumaric acid

We used SEM to explore the potential direct and indirect relationships between coumaric acid and various soil environmental factors affecting tobacco cultivation. Our analysis revealed that soil pH is a key factor influencing microbial diversity, and coumaric acid stress has a direct negative impact on soil pH. Based on this theoretical analysis and our findings, we propose that coumaric acid lowers soil pH, inducing a shift in the soil microbial community structure from bacteria-dominated to fungi-dominated. This shift also impacts soil enzyme activity, with bacterial activity being the most affected. This aligns with previous research (Choma et al., 2020), which demonstrated that bacterial community changes significantly influence soil enzyme activity during the gradual acidification of soil, whereas fungal communities seem to have a lesser effect on enzyme activity. The reduction in soil enzyme activity inevitably leads to imbalances in soil nutrients and stress tolerance, which in turn significantly inhibits tobacco growth. Additionally, coumaric acid stress caused a significant increase in leaf MDA levels, indicating enhanced lipid peroxidation of cell membranes (Kumar et al., 2023), further suppressing plant growth.

## 5 Conclusions

Coumaric acid has the strongest direct allelopathic effect among the six phenolic acids with cumulative effects. Exogenous addition of coumaric acid affects soil pH, shifting the soil microbial community from bacteria-dominated to fungi-dominated, while reducing the abundance of bacteria related to nutrient utilization and fungi related to disease resistance. This leads to a decrease in soil enzyme activity and ultimately inhibits tobacco growth. Coumaric acid also directly inhibits tobacco growth by increasing leaf MDA levels. Our findings highlight the significant role of coumaric acid in tobacco continuous cropping obstacles and reveal its underlying mechanisms. In future efforts to mitigate continuous cropping obstacles in tobacco cultivation, attention should be paid to monitoring coumaric acid levels, with potential strategies focusing on coumaric acid elimination, soil pH regulation and enhancing plant stress tolerance.

## Data Availability

The raw data were uploaded to the Sequence Read Archive (SRA) of the National Center for Biotechnology Information (NCBI) under the accession IDs PRJNA1135263 and PRJNA1135318.
