# Supplementary material for: Accumulation of coumaric acid is a key factor in tobacco continuous cropping obstacles
Source: Front Plant Sci. 2024 Oct 28;15:1477324. doi: 10.3389/fpls.2024.1477324 (PMC11552174; doi:10.3389/fpls.2024.1477324)
Supplement: Supplementary file 1 [file DataSheet1.docx]

Supplementary Material

## 1. Supplementary Figures


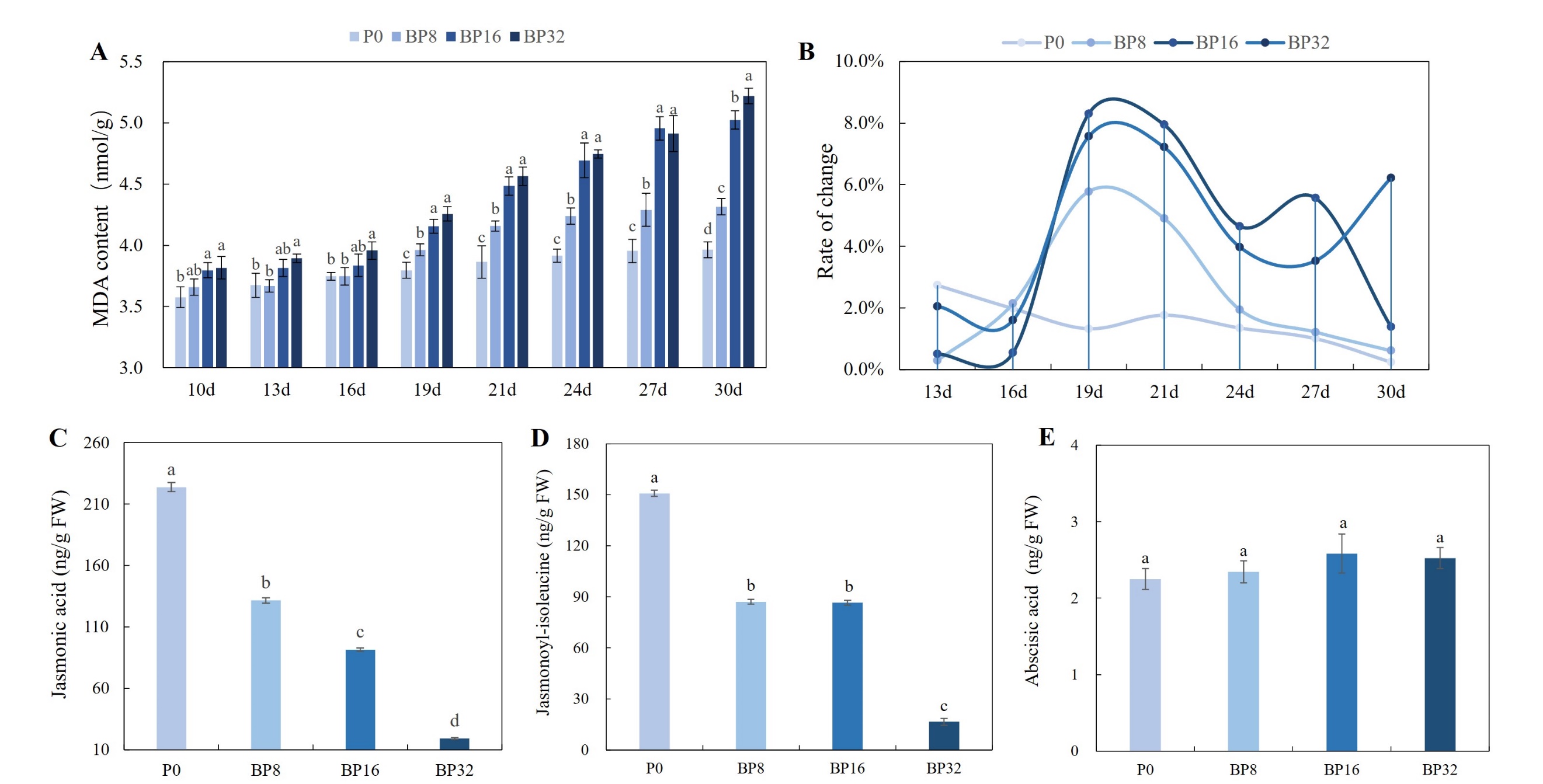


**Supplementary Figure 1.** Physiological indicators of tobacco leaves and roots under coumaric acid stress. (A) MDA content in leaves at various time points across various treatments. (B) Rate of change in MDA content in leaves over time across various treatments. (C–E) Levels of JA, JA-Ile, and ABA in the roots across various treatments. FW: fresh weight. Different letters between the treatments indicate significant difference at *P* ≤ 0.05.


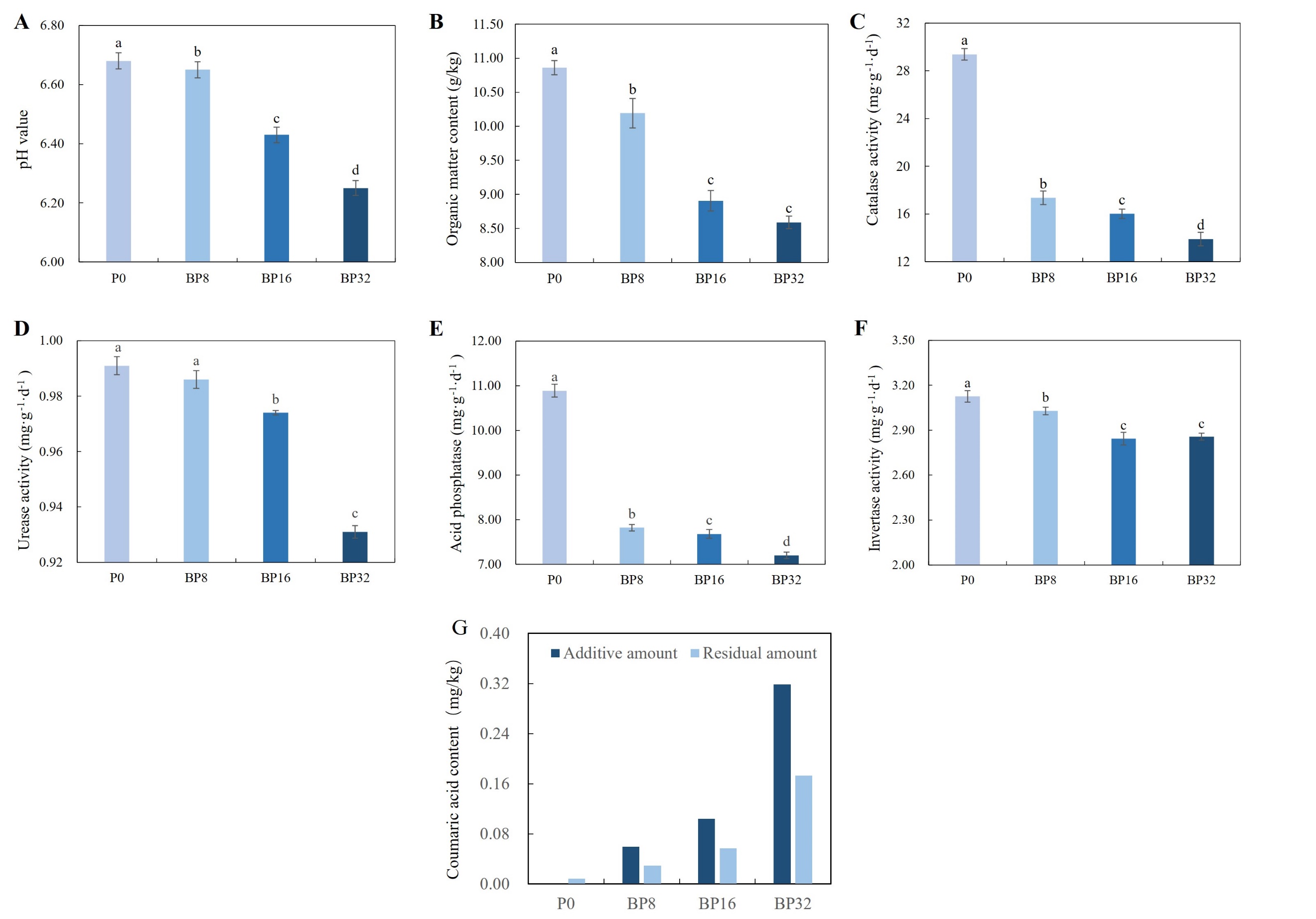


**Supplementary Figure 2.** Analysis of soil chemical properties, enzyme activity changes and residual coumaric acid under coumaric acid stress.

## Supplementary Tables

**Supplementary Table 1.** Soil chemical properties and phenolic acids content.

| **Chemical properties** | **Content** |  | **Phenolic acids** | **Content** |
| --- | --- | --- | --- | --- |
| pH value | 6.72 |  | Phloroglucinol (mg/kg) | 0.0136 |
| Organic matter (g/kg) | 9.43 |  | Coumaric acid (mg/kg) | <LOD |
| Total nitrogen (g/kg) | 1.86 |  | *p*-hydroxybenzoic acid (mg/kg) | <LOD |
| Total phosphorus (g/kg) | 2.84 |  | Vanillic acid (mg/kg) | <LOD |
| Total potassium (g/kg) | 5.32 |  | Vanillin (mg/kg) | 0.0083 |
| Hydrolyzable nitrogen (mg/kg) | 845 |  | Ferulic acid (mg/kg) | 0.0089 |
| Available phosphorus (mg/kg) | 90.25 |  | Benzoic acid (mg/kg) | 0.0078 |
| Available potassium (mg/kg) | 1986 |  | Cinnamic acid (mg/kg) | <LOD |
|  |  |  | Phthalic acid (mg/kg) | <LOD |
|  |  |  | Salicylic acid (mg/kg) | <LOD |

**Supplementary Table 2.** Setting conditions of manual climate box.

| **Time** | **Temperature (℃)** | **Humidity (%RH)** | **Illumination (Lx)** |
| --- | --- | --- | --- |
| 08:00 | 20 | 50 | 5000 |
| 09:00 | 25 | 50 | 6500 |
| 10:00 | 25 | 50 | 8000 |
| 11:00 | 25 | 50 | 9000 |
| 12:00 | 30 | 50 | 10000 |
| 13:00 | 30 | 50 | 10000 |
| 14:00 | 30 | 50 | 10000 |
| 15:00 | 30 | 50 | 9000 |
| 16:00 | 30 | 50 | 8000 |
| 17:00 | 30 | 50 | 6500 |
| 18:00 | 25 | 50 | 5000 |
| 19:00 | 25 | 60 | 0 |
| 20:00 | 25 | 60 | 0 |
| 21:00 | 20 | 60 | 0 |
| 22:00 | 20 | 60 | 0 |
| 23:00 | 20 | 60 | 0 |
| 24:00 | 15 | 60 | 0 |
| 01:00 | 15 | 60 | 0 |
| 02:00 | 15 | 60 | 0 |
| 03:00 | 15 | 60 | 0 |
| 04:00 | 15 | 60 | 0 |
| 05:00 | 15 | 60 | 0 |
| 06:00 | 20 | 60 | 0 |
| 07:00 | 20 | 60 | 0 |

**Supplementary Table 3.** Effects of exogenously added coumaric acid on tobacco growth.

| **Treatment** | **Height (cm)** | **Stem** **girth (cm)** | **Leaf number (leaf)** | **Root weight (g)** |
| --- | --- | --- | --- | --- |
| P0 | 15.29±0.39a | 2.32±0.06a | 6.77±0.17a | 4.06±0.10a |
| BP8 | 13.30±0.30b | 2.04±0.05b | 4.85±0.11b | 3.59±0.08b |
| BP16 | 10.17±0.18c | 1.57±0.03c | 3.93±0.07c | 2.75±0.05c |
| BP32 | 8.31±0.14d | 1.37±0.02d | 3.91±0.07c | 2.39±0.05d |

Note: Values were the mean ± SD of 3 replicates. Different letters between the treatments indicate significant difference at *P* ≤ 0.05.
